# Suppressing miRNA-15a/-16 expression by interleukin-6 enhances drug-resistance in myeloma cells

**DOI:** 10.1186/1756-8722-4-37

**Published:** 2011-09-22

**Authors:** Mu Hao, Li Zhang, Gang An, Weiwei Sui, Zhen Yu, Dehui Zou, Yan Xu, Hong Chang, Lugui Qiu

**Affiliations:** 1 State Key Laboratory of Experimental Hematology, Institute of Hematology & Blood Diseases Hospital, Chinese Academy of Medical Science & Peking Union Medical College Tianjin China; 2 West China Hospital, Sichuan University. Blood Section, Chengdu, Sichuan, China; 3 Department of Laboratory Hematology, University Health Network, University of Toronto, Canada

## Abstract

The bone marrow microenvironment facilitates the survival, differentiation, and proliferation of myeloma (MM) cells. This study identified that microRNA-15a and -16 expressions tightly correlated with proliferation and drug sensitivity of MM cells. miRNA-15a/-16 expression in MM cells was significantly increased after treatment with cytotoxic agents. The interaction of bone marrow stromal cells (BMSC) with MM cells resulted in decreased miRNA-15a/-16 expression and promoted the survival of the MM cells. Interleukin-6 (IL-6) produced by BMSCs suppressed the expression of miRNA-15a and 16 in a time- and dose- dependent pattern, with the suppression on miRNA-15a being more significant than on miRNA-16. miRNA-15a-transfected MM cells were found to be arrested in G1/S checkpoint, and the transfected MM cells had decreased growth and survival. In conclusion, our data suggest that via suppressing miRNA-15a and -16 expressions, IL-6 secreted by BMSCs promotes drug-resistance in myeloma cells.

## To the Editor

**Figure 1:**
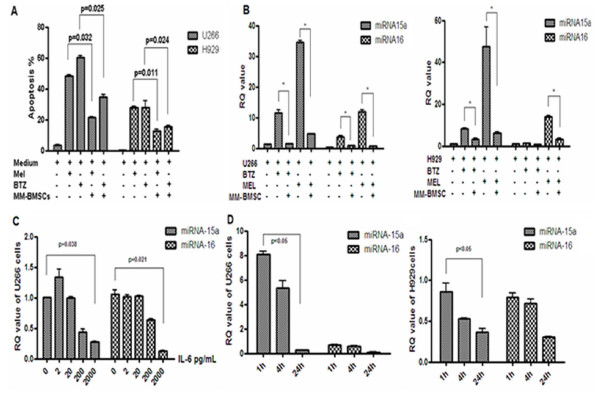
**Bone marrow stromal cells derived from myeloma patients (MM-BMSCs) suppress apoptosis and miRNA-15a/-16 expression in MM cells**. (A) MM-BMSCs inhibited apoptosis of MM cells induced by cytotoxic agent. (B) Stem-loop RT-PCR assay showed that miRNA-15a/-16 expression in MM cells was significantly increased by melphalan and bortezomib treatment. When MM cells were co-cultured with MM-BMSCs, miRNA-15a/-16 expression in MM cells was suppressed. (C & D) IL-6 decreased miRNA-15a/-16 expression in U266 and NCI-H929 cells in a time- and dose- dependent pattern.

Multiple myeloma (MM) is an incurable plasma cell malignancy [1-3]. Binding of MM cells to bone marrow stromal cells (BMSCs) promotes the growth, survival, metastasis and drug resistance of the MM cells. The molecular bases of MM progression and drug resistance remain incompletely understood [4,5]. In this study, apoptosis analysis by flow cytometry showed that BMSCs protect U266 and NCI-H929 myeloma cells from apoptosis induced by melphalan and bortezomib. (Figure 1A). IL-6 and VEGF are critical growth factors for myeloma cells. Both are mainly produced by BMSCs [6-8]. By ELISA analysis, we found that the level of IL-6 and VEGF secreted in the supernatant of BMSCs derived from MM patient (MM-BMSCs) was significantly higher (188.8+9.4 pg/mL and 1497.2+39.7 pg/mL, respectively) than that of normal BMSCs (115.0+15.1 pg/mL and 1239.0+21.1 pg/mL, respectively; p < 0.05).

microRNA -15a and -16 are located on chromosome 13, an area commonly deleted in MM. Deletion of chromosome 13 predicts a significantly reduced survival in patient with MM [9-11]. We thus focused on the functions of miRNA-15a and -16. We found that miRNA-15a/-16 expression in MM cells was significantly increased under melphalan and bortezomib treatment (Figure 1B). Moreover, dexamethasone sensitive MM cell line, MM1S, expressed higher level of miRNA-15a than the resistant MM1R. miRNA-15a expression in MMIS and MM1R was 909.73 ± 7.12 and 134.88 ± 19.85 (p < 0.01), respectively, and miRNA-16 expression in those cells was 9.83 ± 2.01 and 9.20 ± 3.81 (p > 0.05), respectively. Interestingly, the interaction of MM cells with MM-BMSCs inhibited miRNA-15a and -16 expressions in MM cells. (Figure 1B) IL-6 secreted by MM-BMSCs decreased expression of miRNA-15a and -16 in myeloma cells in a time- and dose- dependent pattern. (Figure 1C,D) The suppression on miRNA-15a was more significant than on miRNA-16 in myeloma cells. Previous study has identified cyclinD1, cyclinD2 and CDC25A as the targets of miRNA-15a [12]. Our data further showed that miRNA-15a-transfected MM cells were arrested in G1/S checkpoint. The over-expression of miRNA-15a inhibited growth and survival of the transfected MM cells.

In conclusion, this study identified that microRNA-15a and -16 expressions correlated well with proliferation and drug sensitivity of MM cells. MM-BMSCs enhanced the survival of the MM cells and protected them from drug-induced apoptosis by suppressing miRNA-15a/-16 expression. IL-6 secreted by the MM-BMSCs plays a pivotal role in this process.

## List of Abbreviation

MM: multiple myeloma; BMSCs: bone marrow stromal cells; IL-6: interleukin 6; VEGF: Vascular-Endothelial Growth Factor; ELISA: enzyme-linked immunosorbent assay

## Authors' contributions

MH provided the concept and design of the study, acquisition of data, analysis and interpretation of data, drafting the manuscript; L Zh and GA performed myeloma cell Stem-loop RT-PCR assay; WWS, DHZ collected samples from myeloma patients; ZY and YX assisted in data collection; HC and LGQ revised the manuscript and gave final approval of the version to be submitted. All authors have read and approved the final manuscript.

## Conflicts of Interests

The authors declare that they have no competing interests.
